# EXPLORING THE DIAGNOSTIC AND THERAPEUTIC JOURNEY OF PRIMARY PROGRESSIVE APHASIA: A CASE STUDY FROM A RURAL SETTING

**DOI:** 10.1093/geroni/igae098.3076

**Published:** 2024-12-31

**Authors:** Abigail Avendaño Villaseñor, Ladan Ghazi Saidi

**Affiliations:** Universidad Autónoma de Occidente, Culiacan, Sinaloa, Mexico; University of Nebraska Kearney, Kearney, Nebraska, United States

## Abstract

Primary Progressive Aphasia (PPA) is a young-onset neurological disorder that affects a person’s language, behavior, and executive function abilities, caused by a focal degeneration of the frontal and temporal lobes in the brain. It is considered a frontotemporal dementia (FTD) due to the neuroanatomical areas it affects and the similar etiology it has with other neurodegenerative disorders. Differential diagnosis is difficult for health professionals due to the recent description of clinical variants, and the limited options for clinical treatment. In this cross-sectional and descriptive study, we reviewed the case of a 76-year-old male patient diagnosed with agrammatic primary progressive aphasia (nfvPPA) who lives in a rural area in Nebraska, living with his caregiver. The objective of this case study was to analyze the difficulties experienced by the patient before and after he was diagnosed with nfvPPA, as well as his current progress with treatment. We identified the main challenges they encountered during the diagnostic and therapeutic processes were: a delayed differential diagnosis, inadequate communication from practitioners, inefficient interprofessional communication, and limited treatment/management options. In addition, they had limited access to educational and support group resources, a lack of intervention implementation, failed efforts in the follow-up of treatment by each care provider, and difficult access to health care services. Our findings highlight the necessary changes needed in the management of patients through a collaborative model, to provide sufficient and accessible resources to navigate this diagnosis, and the importance of following up on the therapeutic progress of patients.

